# Satellites may underestimate rice residue and associated burning emissions in Vietnam

**DOI:** 10.1088/1748-9326/aa751d

**Published:** 2017-08-10

**Authors:** Kristofer Lasko, Krishna P Vadrevu, Vinh T Tran, Evan Ellicott, Thanh T N Nguyen, Hung Q Bui, Christopher Justice

**Affiliations:** 1 Department of Geographical Sciences, University of Maryland, College Park, MD 20742, United States of America; 2 Earth Science Office, NASA Marshall Space Flight Center, Huntsville, AL, United States of America; 3 Faculty of Information Technology, Hanoi Pedagogical University 2, Vinh Phuc, Viet Nam; 4 University of Engineering and Technology, Vietnam National University Ha Noi, Ha Noi, Viet Nam

**Keywords:** Rice straw, PM_2.5_, GFED, REAS, biomass burning, southeast Asia, bottom-up

## Abstract

In this study, we estimate rice residue, associated burning emissions, and compare results with existing emissions inventories employing a bottom-up approach. We first estimated field-level post-harvest rice residues, including separate fuel-loading factors for rice straw and rice stubble. Results suggested fuel-loading factors of 0.27 kg m^−2^ (±0.033), 0.61 kg m^−2^ (±0.076), and 0.88 kg m^−2^ (±0.083) for rice straw, stubble, and total post-harvest biomass, respectively. Using these factors, we quantified potential emissions from rice residue burning and compared our estimates with other studies. Our results suggest total rice residue burning emissions as 2.24 Gg PM_2.5_, 36.54 Gg CO and 567.79 Gg CO_2_ for Hanoi Province, which are significantly higher than earlier studies. We attribute our higher emission estimates to improved fuel-loading factors; moreover, we infer that some earlier studies relying on residue-to-product ratios could be underestimating rice residue emissions by more than a factor of 2.3 for Hanoi, Vietnam. Using the rice planted area data from the Vietnamese government, and combining our fuel-loading factors, we also estimated rice residue PM_2.5_ emissions for the entirety of Vietnam and compared these estimates with an existing all-sources emissions inventory, and the Global Fire Emissions Database (GFED). Results suggest 75.98 Gg of PM_2.5_ released from rice residue burning accounting for 12.8% of total emissions for Vietnam. The GFED database suggests 42.56 Gg PM_2.5_ from biomass burning with 5.62 Gg attributed to agricultural waste burning indicating satellite-based methods may be significantly underestimating emissions. Our results not only provide improved residue and emission estimates, but also highlight the need for emissions mitigation from rice residue burning.

## Introduction

Crop residue burning is an important source of greenhouse gases and aerosols (Streets *et al* 2003, Crutzen and Andreae 1990). The burning of crop residues contributes to at least 34% of global biomass burning emissions (Streets *et al* 2004). While these and other studies provide useful general estimates, analyses need to be region-specific to enable emissions mitigation. Of the different crop residues, rice residues are prevalently burned in South/Southeast Asian countries in addition to forest biomass burning (Streets *et al* 2003, Biswas *et al* 2015).

Rice (*Oryza sativa*) is the staple crop for livelihood in Southeast Asia and more specifically, Vietnam. During 2015, Vietnam produced 45.2 million metric tonnes of rice with most production in the Mekong River Delta (57%) and the Red River Delta (15%) (Vietnam Government 2015). The Red River Delta is home to Hanoi, the capital of Vietnam which outside of the immediate downtown area, exhibits a mosaic landscape dominated by paddy rice, small-holder farms, and plantations, all intermixed amongst a growing peri-urban area (Pham *et al* 2015). Thus, in Hanoi, many residential and commercial areas are not only impacted by urban emissions, but also by smoke from rice residue burning. Studies have attributed crop residue burning to local and regional impacts including long-range transport with effects persisting for weeks or months impacting air quality, atmospheric chemistry, weather, and biogeochemical cycles (Badarinath *et al* 2007, 2009, Vadrevu *et al* 2012, 2014, 2015, Cristofanelli *et al* 2014, Reddington *et al* 2014, Ponette-González *et al* 2016, Yan *et al* 2006, Le *et al* 2014). For Hanoi in particular, nocturnal radiation inversions occur during the October rice harvest and burning, greatly enhancing the negative air quality impact of fine-particulate matter emissions (Hien *et al* 2002).

**Figure 1:**
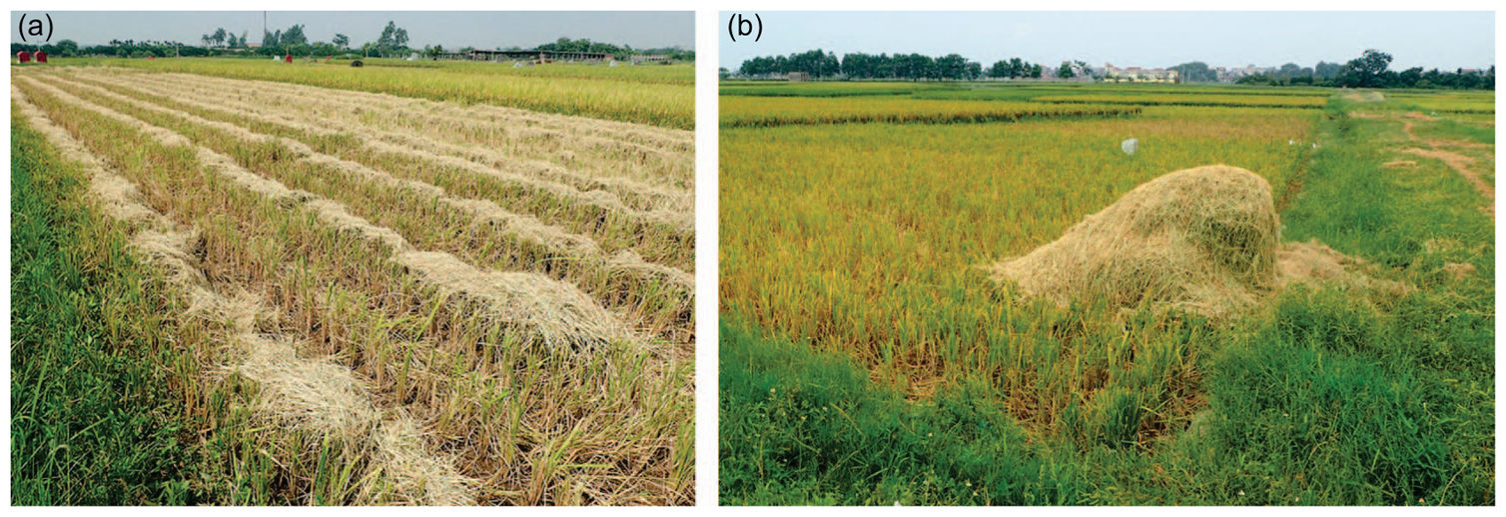
(*a*) Typical machine-harvested field in Hanoi province with dry rice straw laid in neat rows; (*b*) typical rice straw pile prior to burning near Hanoi City.

**Figure 2:**
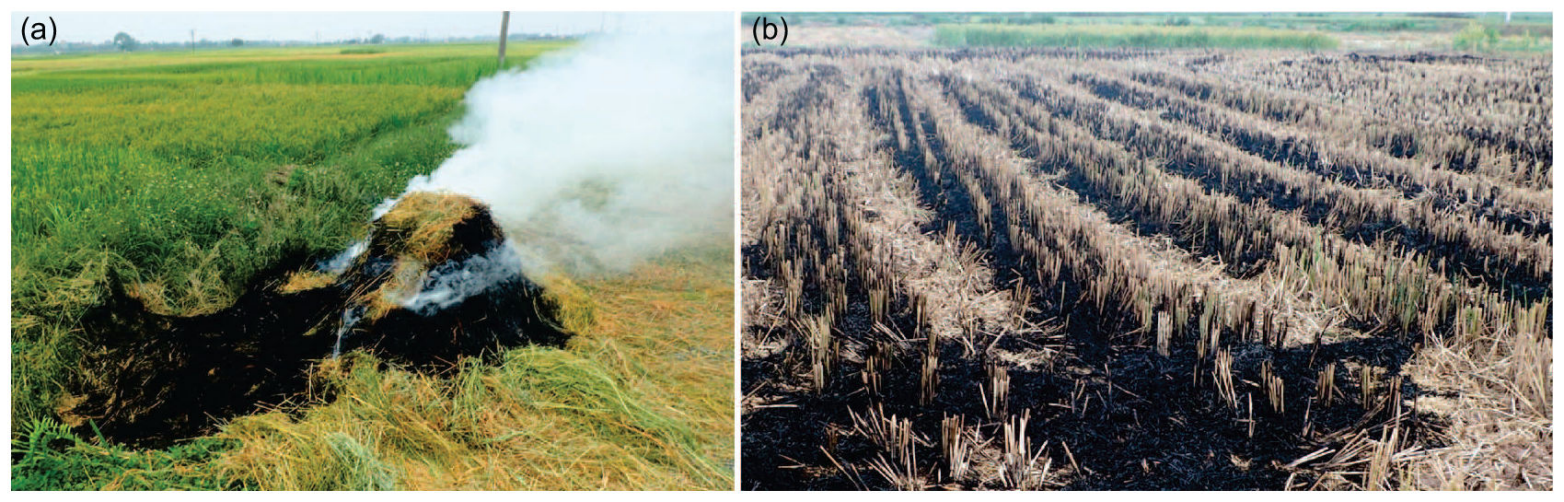
(*a*) Rice straw pile burning. Piles are often burned nearby to the harvested field. (*b*) Typical post-burned rice field in Hanoi Province, Vietnam. Most straw is burned efficiently, however much stubble is left incompletely combusted.

Around Hanoi, the typical paddy rice field size ranges from 150–2280 m^2^ (Patanothai 1996) with an average of 790 m^2^ (*σ =*625 m^2^) (this study). Hanoi is located within the heart of the Red River Delta which is Vietnam’s second largest rice producing hub with over 35% of the land dedicated to rice (Vietnam Government 2015). Rice is routinely irrigated and double-cropped in Hanoi Province with winter rice harvested during June and spring rice harvested during late September–October. After harvest, a large volume of rice straw is left in rows or piles on the field as well as uncut stubble (figures 1(*a*) and (*b*)). In order to prepare the field for the next harvest, farmers routinely burn the residues. Typical burning of a rice straw pile and post-burned field in Hanoi are shown (figures 2(*a*) and (*b*)). Mostly, the straw is burned or reincorporated into the soil while some is used for cattle feed, cook stoves, composting, and mushroom cultivation (Trach 1998, Hong Van *et al* 2014, Duong and Yoshiro 2015, Nguyen 2012, Oritate *et al* 2015). In comparison to the rural areas, suburban areas such as Hanoi typically burn a higher proportion of rice straw as these areas have fewer cattle relying on it for food (Duong and Yoshiro 2015). Thus, with a higher proportion of residue burned in Hanoi, there is amplified impact from emissions. Further, post-harvest rice straw is assumed to have moisture content of about 15% or less, however this varies depending on conditions, and residue structure/density can also have an impact on resulting emissions (Korenaga *et al* 2001, Arai *et al* 2015). In order to estimate emissions impact, accurate bottom-up quantification of residue production and burning is needed.

Earlier studies on estimating emissions from crop residue burning have used agricultural production data, a crop specific residue-to-product ratio, an estimate of the proportion of residue subject to burning, emission factors, and a combustion factor (Streets *et al* 2003, Yevich and Logan 2003, Yan *et al* 2006, Cao *et al* 2008, Gadde *et al* 2009, Kanabkaew and Oanh 2011, Vadrevu and Lasko 2015, Zhang *et al* 2015). While these studies yield insight on emissions estimation, they can be improved by incorporating field-based locally/regionally estimated fuel-loads or emissions factors established from field measurements (Oanh *et al* 2011, Kanokkanjana *et al* 2011, Rajput *et al* 2014, Hong Van *et al* 2014, Arai *et al* 2015). We note that comprehensive province-level field-estimates of rice straw and rice stubble have yet to be generated for northern Vietnam.

Field studies estimating rice straw, stubble, and total post-harvest biomass production are labor intensive and costly. Accordingly, remote sensing with its synoptic and consistent coverage can be used for estimating these factors. In Southeast Asia and Vietnam, forecasting of rice yield or biomass has been done using X-band synthetic aperture radar (SAR) (Gebhardt *et al* 2012, Bouvet *et al* 2014), C-band SAR (Ribbes and Le Toan 1999, Chakraborty *et al* 2005, Lam-Dao *et al* 2009, Inoue *et al* 2014, Karila *et al* 2014), and L-band SAR (Zhang *et al* 2009, Torbick *et al* 2011). Further details on these and related mapping applications are available in recent reviews (Kuenzer and Knauer 2013, Mosleh *et al* 2015, Dong and Xiao 2016). Developing a relationship between field-estimated rice biomass and SAR signal is useful for upscaling field studies to broader regions and time periods. Thus, it contributes to systematic and operational monitoring of rice residue production useful for not only estimating emissions from burning, but also emissions mitigation such as bioenergy generation.

In this study, we develop and assess a straightforward method for efficient and accurate field estimation of rice residue fuel-loading factors for straw, stubble, and total post-harvest biomass. We use these as inputs to calculate resulting potential residue burning emissions for Hanoi, Vietnam and compare results using fuel-loading factors from other studies. Using our fuel-loading factors and those from other studies we upscale our results to the entirety of Vietnam and compare with an existing emissions inventory to assess rice residue burning contribution to total emissions from all sources. We also compare our emission estimates with the satellite-derived estimates from the Global Fire Emissions Database (GFED). We then explore the potential forecasting of rice residue using field and SAR data.

We specifically address the following questions: (1) how much rice straw, stubble, and total post-harvest biomass is left in the field, and how does this compare to other regional studies? (2) What are the resulting rice residue burning emissions for major pollutants (PM_2.5_, CO, and CO_2_) in Hanoi Province? (3) What are the resulting rice residue burning emissions for the entire Vietnam and how do they compare to the existing emissions inventories from different sources? (4) How well does SAR data enable forecasting of post-harvest biomass? We addressed these questions using a field and remote sensing based approach representative of a typical double-cropped rice region in Hanoi Province, Vietnam.

## Datasets and methods

### SAR data

**Figure 3:**
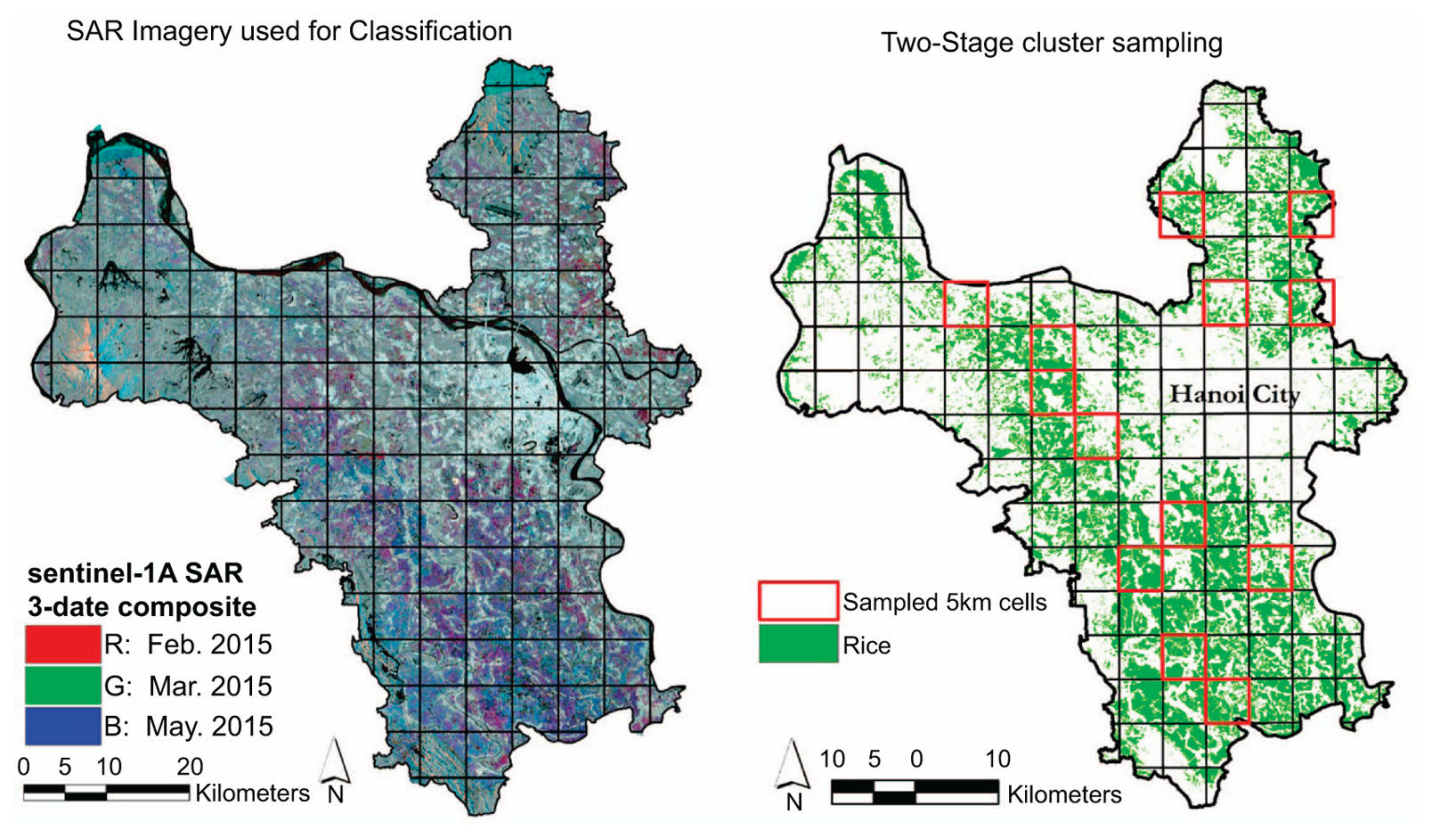
(*a*) A multitemporal subset of our processed SAR data. Areas of purple, pink, and green are indicative of paddy rice; (*b*) Our SAR classified rice map was used to delineate rice and non-rice grid cells for field sampling.

Sentinel-1 carries a 12 m long C-Band SAR yielding a unique ability to penetrate most cloud coverage. Sentinel-1 is a constellation of two satellites including Sentinel-1A with data since October 2014 and Sentinel-1B launched in spring of 2016. The constellation has a repeat-pass of approximately six days for many regions. From the Alaska Satellite Facility, we obtained a single ground-range detected, interferometric wide-swath (IW), dual-polarized image from Sentinel-1A just prior to harvest for Hanoi Province on September 18, 2016. We processed the imagery following detailed guidelines from European Space Agency using the freely-available S1 toolbox (Zuhlke *et al* 2015) including thermal noise reduction, multi-looking azimuthal compressions to 20 m spatial resolution to reduce noise, terrain correction using the SRTM 30 m DEM, speckle filter, and radiometric adjustments to correct for viewing geometry effects (Liang 2005). We first generated a rice map from a full time-series of Sentinel-1A time-series imagery for 2016 using a support vector machine classification method (figures 3(*a*) and (*b*)) and validated it using field data and fine-resolution imagery from Google Earth and field photos; the resulting map had an overall accuracy of 94.3% and approximately 220 000 ha of rice land area. The resulting rice map built upon results from related studies (Torbick *et al* 2017, Nguyen *et al* 2015) and was used to delineate rice from non-rice for field sampling (figure 3(*b*)).

The field data for generating the fuel-loading factors were collected from Hanoi Province and include georeferenced photos of rice and non-rice areas used in our rice area mapping classification training or accuracy assessment. For relating our field-level fuel-loading data to SAR, we used the Sentinel-1 VH-polarized image obtained approximately 2 weeks prior to harvest (September 18, 2016). The field data collection is described in the subsequent section.

### Rice residue and emissions estimation

We developed a field-based approach to estimate total post-harvest rice residues including straw and stubble left in the field for burning. Using two-stage cluster sampling with the validated rice map generated from Sentinel-1A time-series imagery (figures 3(*a*) and (*b*)) we divided the study area into 5 km equal-area grid cells and randomly selected 13 rice-containing cells where four randomly selected machine-harvested fields were sampled in each cell for a total of 52 field samples (C.I. *=*9.6%, C.L. *=*95%) (Sutherland 2006) (figure 3(*b*)). Within each harvested field, we measured the total straw and stubble weight in four randomly selected 0.5 m × 0.5 m quadrats using a Salter-Brecknell digital scale, bag with known weight, and an Extech MO290 moisture device for relative moisture content. Stubble was cut at the base before weighing, and the weight of the bag was subtracted from the measurements. We also collected ancillary data including field length, width, and number of straw rows. We estimated the amount of straw per square meter for a given field based on the following (equation (1)):

(1) $$Q_{\text{rs}}=\frac{(RS_{w}\times (1-RS_{m}))\times SR\times R_{1}}{A}$$

where *Q*_rs_ is the quantity of dry rice straw in kg m^−2^ for a given field, *RS*_w_ is the wet rice straw weight per linear meter of a straw row averaged from the quadrat measurements, *RS*_m_ is the average field-measured relative moisture content, *SR* is the number of straw rows in the field, *R*_l_ is the length of the straw rows in meters, and *A* is the field area in m^2^.

We estimated the amount of rice stubble for a given field based on a similar (equation (2)):

(2) $$Q_{\text{sr}}=SR_{w}\times (1-SR_{m})\times A$$

where *Q*_sr_ is the quantity of dry rice stubble in kg for a given field, *SR*_w_ is the average weight of rice stubble per m^2^ from the quadrat measurements, *SR*_m_ is the average measured rice stubble relative moisture content, and *A* is the area of the field measured in m^2^. The resulting *Q*_sr_ yields a maximum rice stubble fuel-loading factor in kg. We also measured the number of plants in each quadrat for a measure of rice planting density.

Thus, we have three separate fuel-loading factors: straw, stubble, and total post-harvest biomass factors. Using these fuel-loading factors we estimated rice residue burning emissions based on three scenarios (1) all rice straw is burned; (2) all rice straw and stubble are burned; (3) most-likely amount burned based on the previously-mentioned surveys from the literature and our field experience. We compared these estimates to the typical approach used in most studies which relies on a residue-to-product ratio from government data on crop production. In addition, we also compared maximum potential burning estimates derived using fuel-loading factors from two smaller-scale field studies in villages in Thailand, and factors from another small-scale study in Can Tho, Vietnam located in the Mekong Delta.

We calculated the maximum potential emissions from rice residue burning based on the following (equation (3)):

(3) $$E_{a}=A\times FL\times EF_{a}\times PB\times CF$$

where *E_a_* is the maximum potential rice residue burning emissions for a given pollutant in gigagrams, *A* is the paddy rice planted area in hectares based on the Vietnam government statistics, *FL* is the fuel-loading factor or that estimated from crop production data in kg ha^−1^, *EF_a_* is the emissions factor for a given pollutant species in g kg^−1^, *PB* is the proportion of residue subjected to burning (from 0%–100%, i.e. residue left in the field to be burned), and *CF* is the combustion factor indicating the burn completeness (from 0%–100%) for the residues subjected to burning. For all scenarios estimating emissions in Hanoi Province, we selected a combustion factor of 0.8 (Aalde *et al* 2006) as the best available factor representative of croplands in general, the rice area in Hanoi Province based on Vietnam government statistics of 200.8 ha (Vietnam Government 2015), the emission factors for each species: CO 102 g kg^−1^ (±33 g kg^−1^), CO_2_ 1585 g kg^−1^ (±100 g kg^−1^), and PM_2.5_ 6.26 g kg^−1^ (±2.36 g kg^−1^) (Akagi *et al* 2011), the proportion burned as 100% for the maximum emissions scenario, and 50% straw and 10% stubble for the most-likely scenario based on the literature and our field experience (Tran *et al* 2014, Nguyen 2012, Duong and Yoshiro 2015). To estimate the fuel-loading factor for the production-statistics based scenario, we used a regionally-estimated residue-to-product ratio for rice straw of 0.75 (Gadde *et al* 2009b) combined with the production data from the government statistics (Vietnam Government 2015) resulting in a fuel-loading factor of 3803 kg ha^−1^. For the other smaller-scale studies, we used their fuel-loading factors of 3600 kg ha^−1^ (Kanokkanjana *et al* 2011), 5800 kg ha^−1^ (Oanh *et al* 2011), and 3470 kg ha^−1^ (Hong Van *et al* 2014). The latter factor was derived by averaging the reported seasonal fuel-loading factors of 3500 kg ha^−1^ (spring–summer), 4300 kg ha^−1^ (winter–spring), and 2600 kg ha^−1^ (summer–autumn). For each scenario, all factors were held constant except for the fuel-loading factor and proportion burned.

As our study measurements are representative of a typical double-cropped paddy rice landscape and results from (Hong Van *et al* 2014) cover triple cropping in Mekong Delta Vietnam, we up-scaled the results from both studies to estimate contribution of PM_2.5_ emissions from rice residue burning for the entire Vietnam. The rice planted area for 2008 was obtained from Vietnam Statistics Office (Vietnam 2015). We applied the triple cropping factors to the Mekong Delta rice area, and our study’s fuel-loading factors to the rest of the rice area in Vietnam. We compared the resulting rice burning emissions estimates with an existing emissions inventory (regional emission inventory in Asia (REAS)) for the latest available year, 2008 (Kurokawa *et al* 2013). We calculated Vietnam’s total emissions (equation (3)) for different scenarios including the maximum potential emissions (all residue burned), and the most-likely scenarios of straw and stubble burning based on the literature and our field experience suggesting approximately 50% of straw burned and 10% of stubble burned (Nguyen 2012, Hong Van *et al* 2014, Tran *et al* 2014, Duong and Yoshiro 2015, Oritate *et al* 2015).

While comparison of rice residue burning emissions with REAS yields insight into the relative contribution to total PM_2.5_ emissions, this does not address how our field-derived estimates compare with available satellite-derived estimates. The latest available GFED version 4.1s at 0.25 × 0.25 degree was used to derive total biomass burning emissions for Vietnam, GFED also includes the portion contributed from agricultural waste burning (van der Werf *et al* 2010) based on MODIS burned area (Giglio *et al* 2013), and supplemented by small fire burned area (Randerson *et al* 2012). We calculated the emissions for the same year as REAS (2008) using the monthly datasets, and the same emission factors as our field study to maintain consistency. Accordingly, the emissions were derived using the amount of dry matter burned (DM) from GFED and multiplied with the aforementioned emission factors and summed for entire Vietnam.

We also evaluated the accuracy of our field-based straw measurements in eight additional randomly selected fields within Hanoi Province. First, following the same procedure as the initial field data collection we calculated the expected dry straw weight. To calculate the observed dry straw weight, we collected all rice straw within the field and weighed it. After factoring in the weight of the measuring bags and the average moisture content we arrived at an observed dry straw weight. We then compared this to our expected dry rice straw weight (kg m^−2^) estimated from our field calculation (equation (1)). Using these observed and expected rice straw values we calculate an area-weighted root mean square error (RMSE). We also generated a residual plot to check for systematic errors. As the stubble measurement is more straightforward, we assumed the same accuracy as the straw measurement.

In addition to fuel-loading factor error, we also calculated the resulting emissions error rates through the simplified error propagation equations using reported error rates for emission factors, and fuel-loading factors while also accounting for constants with unknown error such as rice area (Harvard 2013).

## Results

### Fuel-loading factors

**Figure 4:**
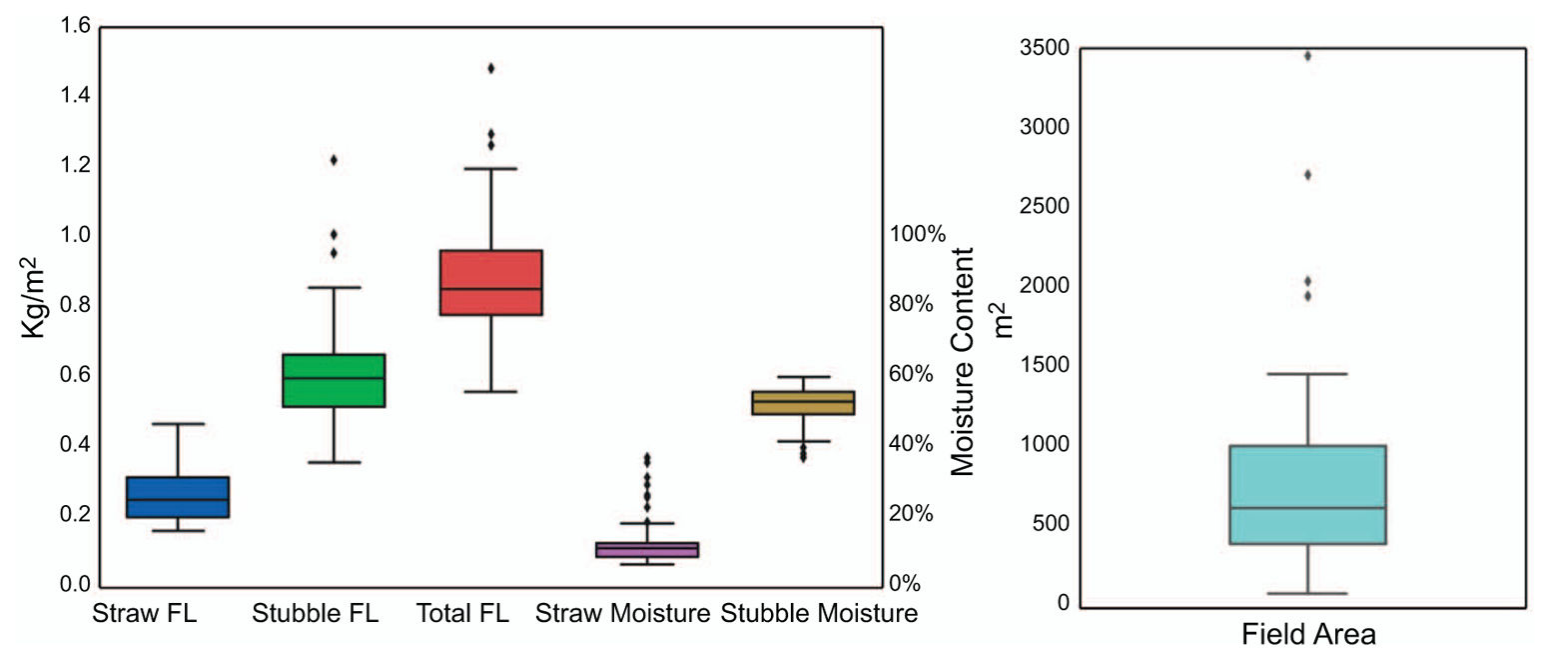
Boxplots highlighting the distribution of field values for area, straw, stubble, and total post-harvest residue in kg m^−2^, as well as straw and stubble moisture content (in percent moisture measured in the field).

Results on field-level distribution of values for rice straw, stubble, total post-harvest biomass, straw moisture content, stubble moisture content, and field area are shown in figure (4). The average fuel-loading factors as measured were: rice straw 0.27 kg m^−2^, rice stubble 0.61 kg m^−2^, and total post-harvest residue 0.88 kg m^−2^; table (1) lists more details including standard deviation and moisture content. The range of standard deviations suggests some spatial variability for the fuel-loading factors and higher ranges for the moisture content attributed to recent rain events. We also found an average field size of 790 m^2^ (*σ =*625 m^2^), and average number of rice plants of 35.1 per m^2^ (*σ =*5.2). Based on the Vietnam government’s rice planted area data for 2015 and our fuel-loading factor, we estimated total straw production for Hanoi Province at 433.7 Gg of dry rice straw. We also estimated total dry rice stubble as 979.9 Gg, and total post-harvest rice biomass as 1413.6 Gg of residue.

**Figure 5:**
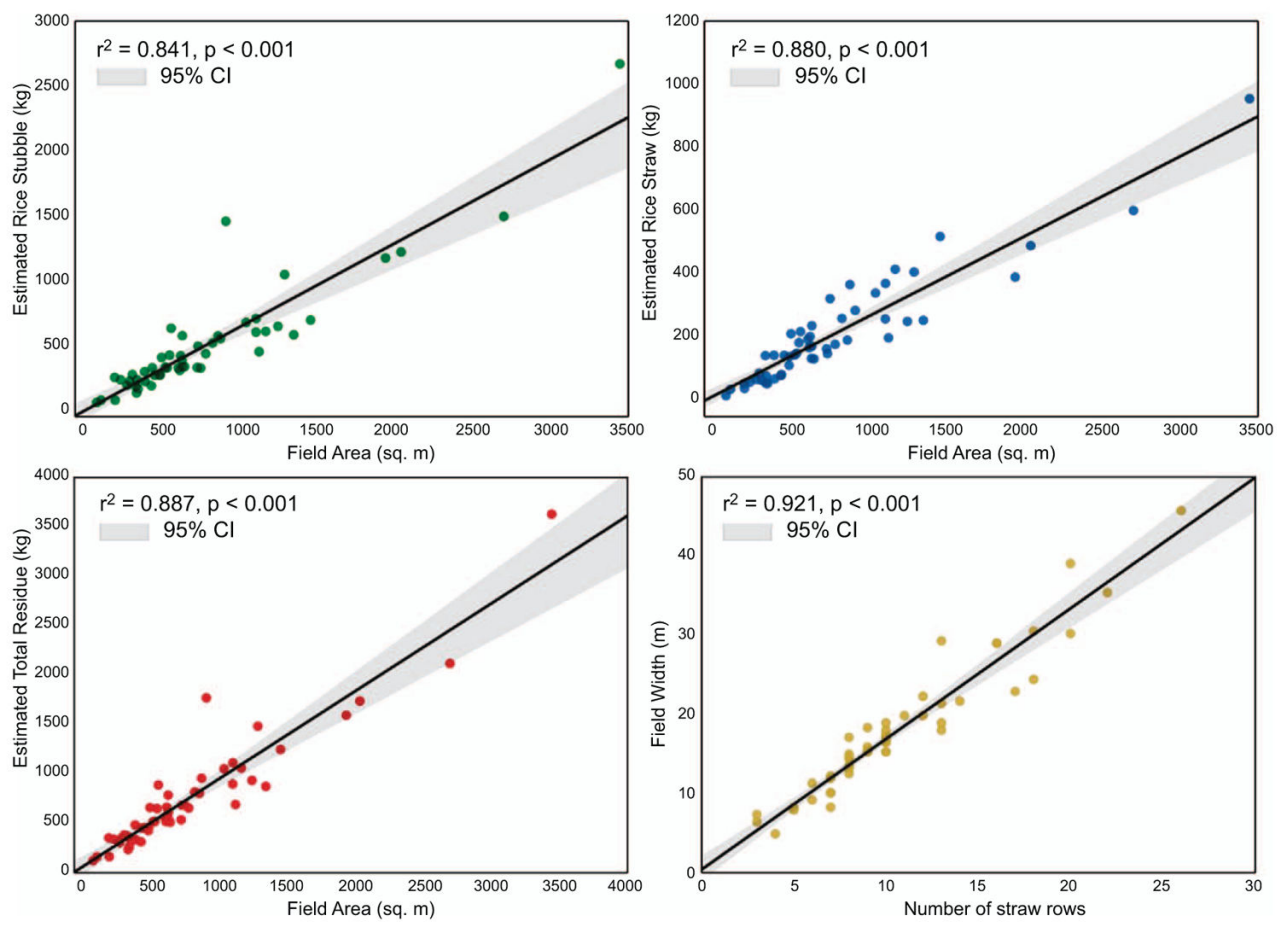
Plots of our field-measured rice residue parameters highlighting a strong relationship between field area and (*a*) rice stubble; (*b*) rice straw; (*c*) total post-harvest rice residue; as well as (*d*) field width and number of rice straw rows per field.

**Figure 6:**
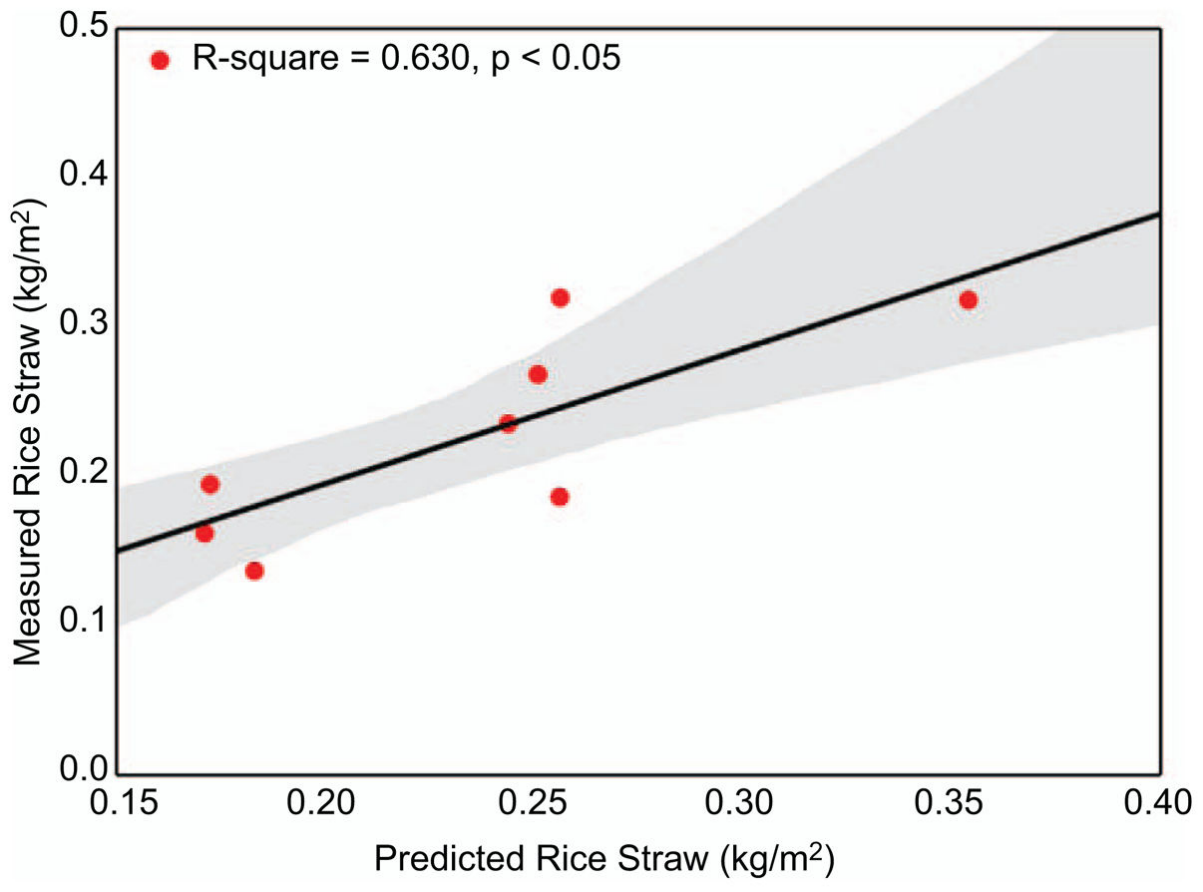
Relationship between the observed and expected rice straw values from the accuracy assessment highlighting a moderately strong relationship.

We observed strong correlations between different field parameters (figures 5(*a*) and (*d*)) i.e. field area and estimated rice stubble (*r*^2^ *=*0.84), field area and estimated rice straw (*r*^2^ *=*0.88), field area and total post-harvest biomass (*r*^2^ *=*0.89) and number of straw rows and field width (*r*^2^ *=*0.92). We also conducted an accuracy assessment of the field-estimated fuel-loading factors. Based on our field measurements of rice straw, we found an RMSE of 0.041 kg m^−2^ between the observed and expected values (equation (1)). After also factoring in error propagation from the moisture content device (3% lab-estimated) and scale (0.4%) we arrived at our final fuel-loading factor estimates; dry rice straw is estimated to be 0.27 kg m^−2^ (±0.033), dry rice stubble at 0.61 kg m^−2^ (±0.076), and total post-harvest biomass at 0.88 kg m^−2^ (±0.083). Further, for our fuel-loading factors we observed a moderate relationship between the observed and expected rice straw values (*r*^2^ *=*0.63, *p* < 0.05) (figure 6) suggesting slight overestimation in our expected rice straw values attributed to field variability including occasional degraded/cut stalk included in measurements.

As compared with the rice straw amount derived from the crop production statistics, our rice straw factor is slightly lower (3803 kg ha^−1^ vs. 2700 kg ha^−1^). However, when incorporating stubble, we account for a total biomass estimate which is not calculated in the production statistics method. We note other studies outside Vietnam found higher fuel-loading factors including 3600 kg ha^−1^ and 5800 kg ha^−1^ in two separate studies in Thailand (Kanokkanjana *et al* 2011, Oanh *et al* 2011), while the other study in the Mekong Delta, Vietnam had a lower average of 3470 kg ha^−1^ (Hong Van *et al* 2014). This wide variation suggests region specific fuel-loading factors are an important base for emissions estimates.

### Emissions estimates

In Hanoi Province, we used our resulting fuel-loading estimates for quantifying rice residue emissions. We used the cropped area data, combustion factor, and emission factors for CO, CO_2_, and PM_2.5_ as described above. We present the emissions for each scenario including: maximum emissions from typical production-statistics, maximum emissions using fuel-load factors from field-based study in Can Tho, Vietnam (Hong Van *et al* 2014), and maximum potential and most-likely emissions using our fuel-load factors of rice straw, and total post-harvest biomass. We also estimated maximum potential emissions using fuel-load factors from two village-level studies in Thailand (Kanokkanjana *et al* 2011, Oanh *et al* 2011) using their respective rice straw fuel-loading factors of 3600 kg ha^−1^ and 5800 kg ha^−1^. Our results suggest maximum emissions ranges for CO (44.24–144.19 Gg), CO_2_ (687–2240 Gg), and PM_2.5_ (2.72–8.85 Gg) in table (2). Based on averaging the residue burning survey data and from our field experience, the most likely proportion burned is 50% straw and 10% stubble. We find the most-likely combined straw and stubble emissions for Hanoi as CO (36.54 Gg), CO_2_ (567.79 Gg), and PM_2.5_ (2.24 Gg). The individual scenario results are presented in table 2 including the error rates for each value in parentheses.

We highlight notable variation between the different scenarios. While all rice stubble is not necessarily burned in the study area, in other regions such as India both the straw and stubble are routinely burned (Gadde *et al* 2009, Gupta *et al* 2001, Vadrevu *et al* 2015). However, many studies estimating emissions from rice residue burning rely on residue-to-product ratios. Without accounting for rice stubble that is actually burnt on field, these and other studies may be underestimating emissions by a factor of about 2.3 (table 2).

We also estimated total rice residue burning emissions for the entire Vietnam during 2008 and compared with the existing emission inventory (Kurokawa *et al* 2013). Based on the most-likely emissions scenario (10% stubble, 50% straw burned), 75.98 Gg of PM_2.5_ are emitted from rice residue burning in Vietnam with stubble accounting for 18.36 Gg and straw 57.62 Gg. The total rice residue burning accounts for 12.8% of Vietnam’s total PM_2.5_ emissions and is the 2nd highest PM_2.5_ combustion source after fuelwood burning. However, if all rice residue is burned, rice emissions could result in up to 36.49% of total PM_2.5_ emissions. We also add our emissions estimates to the original total from the emissions inventory of 519.81 Gg (Kurokawa *et al* 2013) and arrive at new maximum of 818.50 Gg and most-likely total PM_2.5_ emissions of 595.79 Gg for the entire Vietnam. Table (3) contains the factors used for estimation, and the individual scenario results including error estimates for residue burning emissions.

**Figure 7:**
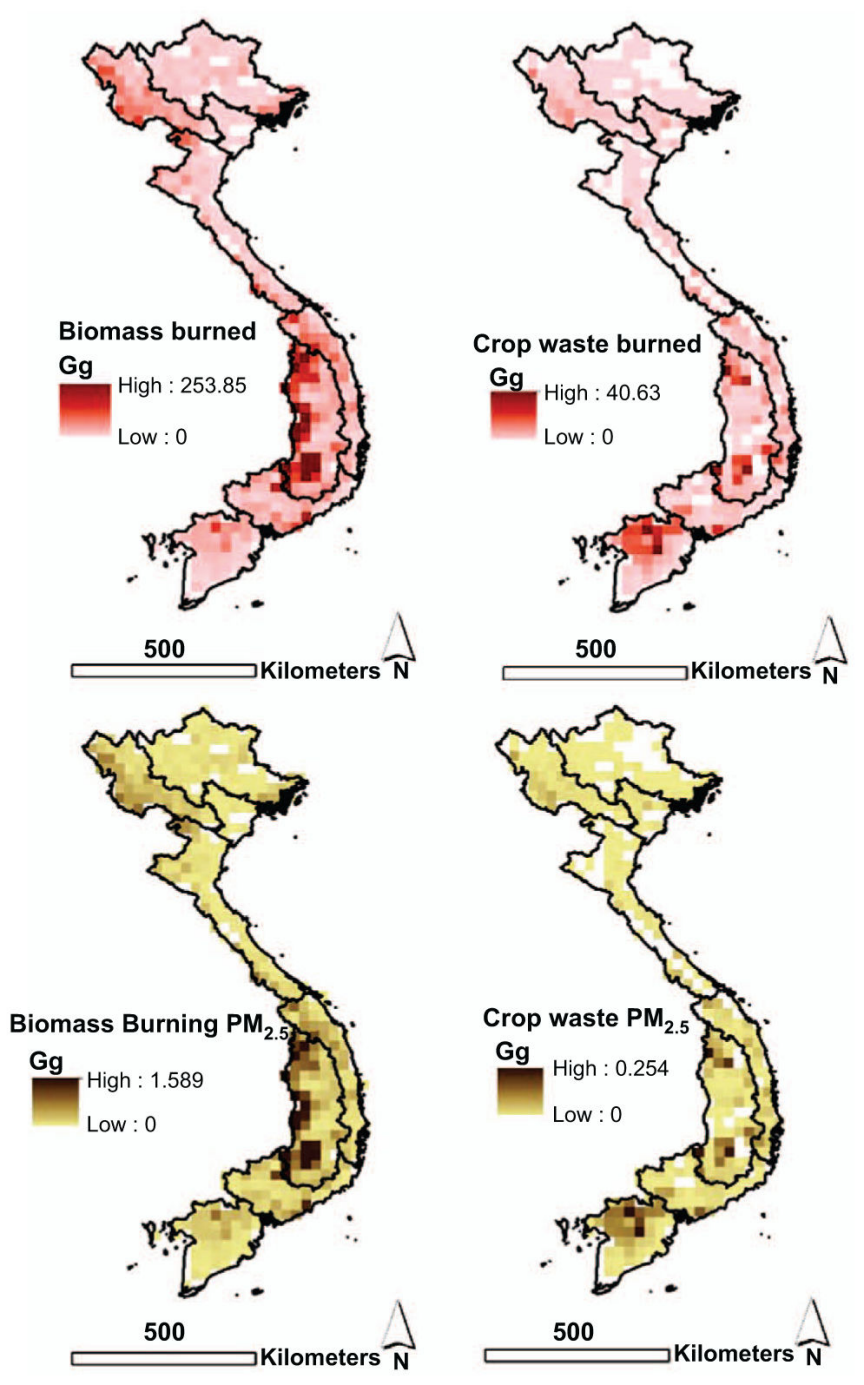
GFED-based spatial variation with region boundaries overlaid for: total biomass burned, crop waste amount burned, and resulting PM_2.5_ emissions from the same.

GFED-derived PM_2.5_ total biomass burning emissions suggested 42.56 Gg (±16.0 Gg) with 5.62 Gg (±2.1 Gg) attributed to agricultural waste burning for the entire Vietnam. Accordingly, when agricultural waste burning emissions are compared with our field-derived residue burning emission estimates this suggests GFED underestimation by a factor of 13.5. Even if all biomass burning emissions (42.56 Gg) were attributed to residue burning, this would still be less than our field-derived estimates by a factor of 1.8. We also highlight the region-level amount of biomass burning, amount of crop residue burning, and associated emissions in table (4) suggesting the highest fraction of crop waste burning to occur in the Mekong River Delta and Red River Delta where rice is the predominant land cover. The spatial variation of biomass burning and emission estimates is highlighted in figure (7).

### SAR data and biomass relationship

**Figure 8:**
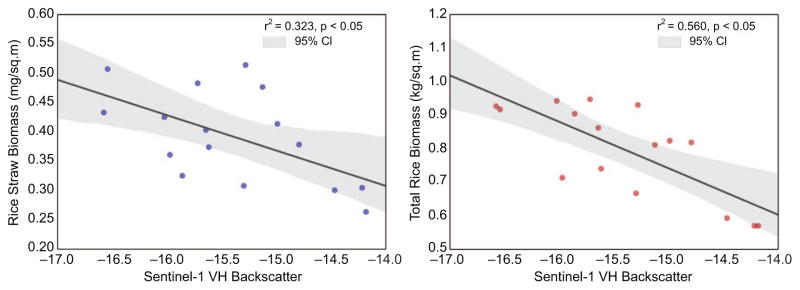
Highlights the relationship of the Sentinel-1 VH backscatter in decibels with (*a*) rice straw biomass measured in the field; and (*b*) total post-harvest rice biomass measured in the field.

To evaluate the relationship of SAR backscatter with rice straw and total post-harvest biomass, we selected a single Sentinel-1A image just prior to harvest for the Hanoi Province. We found a moderately-weak relationship between the SAR backscatter and field-measured rice straw (*r*^2^ *=*0.323, *p* < 0.01), while a moderate relationship was observed with the total rice biomass (*r*^2^ *=*0.560, *p* < 0.01) (figures 8(*a*) and (*b*)). We observed a negative, linear relationship suggesting fields with lower backscatter values prior to harvest have more post-harvest biomass. The relationship is promising, however it needs more refinement in order to be useful for estimating rice biomass and rice straw production prior to harvest. Other studies have found good results using SAR to estimate different rice field properties, biomass, or yield (Lam-Dao *et al* 2009, McNairn and Brisco 2004, Ribbes and Le Toan 1999, Wiseman *et al* 2014, Paloscia *et al* 1999, Bouvet *et al* 2014, Erten *et al* 2016, Inoue *et al* 2014, Satalino *et al* 2015).

## Discussion

In this study, we focused on measuring residue amounts from machine-harvested fields. With the increasing urbanization, infrastructure, wealth, and interconnectedness, we anticipate most fields to switch from hand-harvesting to machine-harvesting (Nguyen *et al* 2016). Hand-harvested fields are typically cut slightly higher from the ground, thus they have more stubble and less straw to potentially burn. Thus as machine-harvested fields become increasingly prevalent, residue burning emissions will be exacerbated, as it is more difficult to collect straw after machine harvest (Nguyen *et al* 2016). Some studies ignore the stubble that is left on the ground, thus underestimating total residue and resulting emissions. However, as undertaken in this study we quantify fuel-loading factors for straw and stubble which provides an improved assessment of rice residue burning emissions, as other studies often ignore the stubble factor. Our estimates incorporating fuel-loading factors for rice straw, rice stubble, and total rice biomass were useful in refining emissions in the Hanoi Province and the entirety of Vietnam. The results can be extended to similar small-holder rice production lands. We note that farmers may burn rice straw either in a pile or in an open-manner including the stubble and straw. Thus, in addition to variable fuel-loads from different harvest methods, the orientation of the rice straw and piling might play a role in the combustion, moisture content, emissions factor, and resulting emissions impact; some of which have been explored, but further quantification is needed (Arai *et al* 2015, Heinsch *et al* 2016). Understanding the variation inresidue management practices and how they impact emissions, may provide useful additional information on emissions mitigation.

Although the total estimated rice burning emissions may be relatively small as compared to other combustion and non-combustion sources (Kurokawa *et al* 2013), rice residue burning in Hanoi is practiced two times per year, amplifying and temporally-concentrating the impact from burning. Accordingly, the air quality in Hanoi is frequently degraded as seen by local haze, and a high air quality index (Nguyen *et al* 2015). Thus, emissions mitigation from rice residue burning is one critical aspect for improving air quality and human health.

Uncertainty with regard to different aspects of fuel-loading and emissions are important to characterize. Further, due to limited availability of data we highlight some future needs which could improve upon the rice stubble and straw burning emissions estimates. Accordingly, improvements could be made by: using separate combustion factors and emission factors for straw and stubble, deriving improved emissions factors specific to rice in both the Red River Delta and Mekong River Delta, improved mapped rice areal estimates, region/crop specific combustion factors for straw and stubble, and machine versus hand-harvested fuel-loading factor comparisons. We note the government estimates of rice land area for Hanoi are different: government statistics (201 000 ha) versus mapped area (220 000 ha). Accordingly, these variations could have significant impact on resulting emissions estimates, especially once aggregating results to the national scale and accounting for both seasons.

Notably low GFED emission estimates could be attributed to a variety of factors such as prevalent cloud cover impacting satellite observations (Wilson and Walter 2016), small size of agricultural fires in Vietnam, ephemeral agricultural fires, active field management, or burning after MODIS overpass time. All of these factors may lead to satellite-based underestimation of fires and resulting emissions regardless of the fire detection algorithm strength in GFED or any other emission database. We further note that the GFED agricultural waste burning data includes emissions from all agricultural sources, yet the emissions are still notably lower than rice residue burning alone. However, these results are presented with the caveat that the field-based emission estimates could be improved through refinements mentioned in the limitations section. We also note for GFED emissions error rates that emission factor error is included, but not burned area error.

## Conclusion

The first part of our study characterized total post-harvest rice residues for the small-holder rice-dominated province of Hanoi, Vietnam. From the field, we developed separate straw, stubble, and total biomass fuel-loading factors representative of a typical double-cropped rice region of Vietnam. Our results on rice residues suggested relatively higher total post-harvest residue factor than the earlier studies. We used Sentinel-1 C-band SAR data for field sampling of residues and to infer the relationship between post-harvest biomass and SAR. We found a moderately weak relationship between the SAR backscatter and field-measured rice straw, while a moderate relationship was observed with the total rice biomass. These results are promising, however, more advanced modelling might be necessary for forecasting the post-harvest biomass using SAR data. We note that not all field data was available for SAR modeling due to GPS or geolocation issues.

Using field data from multiple studies, we then estimate residue burning emissions. We found that rice residue burning accounts for about ~13% of total PM_2.5_ emissions in Vietnam, and is the second largest PM_2.5_ source after fuelwood burning. We compared GFED-derived biomass burning emissions and crop waste burning emissions with our field-derived residue burning emissions. We found a likely notable underestimation by GFED for Vietnam by a factor of over 13. These results suggest a need for improved satellite-derived estimates. Further, emissions mitigation in this sector may be more critical than previously known and is increasingly important for health and air quality concerns in Hanoi, Vietnam.

## Figures and Tables

**Table 1:** Field-derived fuel-loading factors for rice straw, rice stubble, and combined total post-harvest rice residues. Uncertainty: rice straw (±0.033), rice stubble (±0.076), total residue (±0.083) kg m^−2^, moisture content (±3%).

	Rice Straw	Rice Stubble	Total Residue
Average Dry Fuel Load	0.27 kg m^−2^	0.61 kg m^−2^	0.88 kg m^−2^
St. Dev. Dry Fuel Load	0.08 kg m^−2^	0.16 kg m^−2^	0.19 kg m^−2^
Average Fuel Moisture Content	13.2%	51.5%	32.4%
St. Dev. Fuel Moisture Content	7.3%	5.7%	6.5%

**Table 2:** Potential emissions for Hanoi Province, Vietnam using fuel-loading factors from the literature and our study. Gg *=* gigagrams. Maximum potential emissions assume 100% burned for first five listed scenarios, while the bottom three scenarios are the most-likely emissions based on survey and field data on residue proportion subject to burning.

Scenario	Fuel load (kg ha^−1^)	PM_2.5_ Emissions (Gg)	CO Emissions (Gg)	CO_2_ Emissions (Gg)
Crop Production Stats-based FL	3803	3.824	62.31	968.30
Can Tho, Vietnam straw FL (Hong Van *et al* 2014)	3470 (±830)	3.489 (±7.759)	56.86 (±113.93)	883.51 (±10884)
Can Tho, Vietnam FL (Straw & Stubble)	7330 (±1730)	7.371 (±16.327)	120.10 (±239.53)	1866.32 (±22706)
This study (straw only)	2700 (±330)	2.715 (±5.359)	44.24 (±76.20)	687.46 (±4709)
This study (straw and stubble)	8800 (±830)	8.849 (±17.126)	144.19 (±241.99)	2240.61 (±12662)
This study, most-likely emissions (straw)	2700 (±330)	1.629 (±3.215)	26.54 (±45.72)	412.48 (±2825)
This study, most-likely emissions (stubble)	6100 (±760)	0.613 (±1.219)	10.00 (±17.38)	155.31 (±1126)
This study, most-likely emissions (straw and stubble)	—	2.243 (±5.309)	36.54 (±75.02)	567.79 (±3925)

**Table 3:** Potential emissions from rice residue burning for Vietnam. Comparison with the REAS emission inventory (Kurokawa *et al* 2013) and contribution of rice residues to total PM_2.5_ emissions are also shown. Error estimates for emissions are in parentheses. MRD *=* Mekong River Delta.

Scenario	Rice area Mekong Delta (ha)	Rice area rest of Vietnam (ha)	Fuel load rest of Vietnam (kg ha^−1^)	Fuel load Mekong Delta (kg ha^−1^)	Combustion factor	Proportion burned	PM_2.5_ EF g kg^−1^	Rice burned MRD (Gg)	Rice burned rest of Vietnam (Gg)	Rice residue PM_2.5_ (Gg)	Emissions inventory total PM_2.5_ (Gg)	New total emissions inventory PM_2.5_ (Gg)	Percent of total emissions from rice residue
Entirety of Vietnam: Maximum potential emissions (100% straw and stubble burned)	3858900	3563300	8800	7330	0.8	1	6.26	22628.59	25086.72	298.70 (±87.72)	519.81	818.50	36.49
Entirety of Vietnam: Most likely Scenario (50% straw)	3858900	3563300	2700	3470	0.8	0.5	6.26	5356.15	3848.36	57.62 (±17.76)	519.81	577.43	9.98
Entirety of Vietnam: Most likely Scenario (10% stubble)	3858900	3563300	6100	3867	0.8	0.1	6.26	1193.79	1738.89	18.36 (±5.22)	519.81	538.17	3.41
Entirety of Vietnam: Most likely Scenario (above two combined)	–	–	–	–	–	–	–	6549.94	5587.25	75.98 (±22.97)	519.81	595.79	13.39

**Table 4:** GFED-derived region-wise total biomass burned, total biomass burning PM_2.5_ emissions, crop waste amount burned (derived from GFED subset), crop waste burning PM_2.5_ emissions, as well as the percentage of crop waste burning out of total biomass burning.

Region	Total biomass burned (Gg)	Total biomass burning PM_2.5_ (Gg)	Crop waste burned (Gg)	Crop waste burning PM_2.5_ (Gg)	Contribution from crop waste burning
Central Highlands	3215.18	20.13	258.16	1.62	8.0%
Mekong River Delta	384.43	2.41	277.01	1.73	72.1%
North Central Coast	438.20	2.74	41.54	0.26	9.5%
North East	423.84	2.65	26.60	0.17	6.3%
North West	925.75	5.80	86.21	0.54	9.3%
Red River Delta	29.52	0.18	20.40	0.13	69.1%
South Central Coast	595.82	3.73	91.87	0.57	15.4%
South East	785.76	4.92	96.54	0.60	12.3%
Total	6798.50	42.56	898.33	5.62	13.2%
